# Omentin changes following bariatric surgery and predictive links with biomarkers for risk of cardiovascular disease

**DOI:** 10.1186/s12933-014-0124-9

**Published:** 2014-08-21

**Authors:** Marc Lapointe, Paul Poirier, Julie Martin, Marjorie Bastien, Audrey Auclair, Katherine Cianflone

**Affiliations:** Centre de Recherche de l’Institut Universitaire de Cardiologie & Pneumologie de Québec, Université Laval, Y4332, 2725 Chemin Ste-Foy, Québec, QC G1V 4G5 Canada; Faculté de Pharmacie, Université Laval, Québec, Canada; Faculté de Médecine, Université Laval, Québec, Canada

**Keywords:** Obesity, Weight loss, NT-proBNP

## Abstract

**Background:**

Although no receptor has yet been identified, changes in circulating levels of the adipokine designated as Omentin have been demonstrated in obesity and related comorbidities such as cardiovascular disease, insulin resistance, metabolic syndrome and chronic inflammation.

**Methods:**

Changes in Omentin levels at 1 and 5 days and 6 and 12 months in response to biliopancreatic diversion with duodenal switch bariatric surgery were evaluated, specifically to investigate if changes preceded gain of insulin sensitivity.

**Results:**

Pre-operative plasma Omentin was not different between men (n = 18) vs women (n = 48), or diabetic status but correlated with body mass index (BMI). Altogether, Omentin increased as early as 24-h post-surgery, with changes maintained up to 1-year. Fifty-nine percent of subjects increased Omentin >10% by 24-H following surgery (Omentin_INC_ p < 0.0001), while 18% of subjects decreased (Omentin_DEC_ p < 0.0001), with changes maintained throughout one-year. These two groups had comparable age, sex distribution, diabetes, BMI, waist circumference and fat mass, however Omentin_DEC_ had elevated levels of cardiovascular risk markers; homocysteine (p = 0.019), NT-proBNP (p = 0.006) and total bilirubin (p = 0.0001) while red blood cell (RBC) count was lower (p = 0.0005) over the one-year period. Omentin levels at 1-DAY also correlated with immune parameters (white blood cell count, % neutrophil, % monocytes, % lymphocytes).

**Conclusion:**

Omentin_DEC_ at 1 day following surgery may be a marker of cardiovascular “at-risk” group before weight loss or insulin sensitivity restoration.

## Background

A recently described adipokine, Omentin, has gained interest lately following reports of associations with various comorbidities stemming from obesity such as cardiovascular disease, insulin resistance and metabolic syndrome (reviewed in [1]). Omentin expression varies throughout the body (heart, lungs, ovary and placenta) but its main tissue of production is now considered to be visceral adipose tissue [2,3]. First identified in intestinal Paneth cells, Omentin demonstrates low affinity binding to bacterial cell wall carbohydrates and was thought to be part of early-defense mechanisms in the gut against pathogenic bacteria [4].

In obesity, Omentin levels are decreased and are inversely correlated to body mass index (BMI), waist circumference and leptin concentration [5]. Omentin levels correlate inversely with markers of metabolic syndrome [6–8] and is decreased in patients with heart disease. Omentin has been suggested to be an even better marker than adiponectin of patients at risk for coronary artery disease [9]. Other studies have also shown a significant decrease in Omentin in subjects with demonstrated carotid plaque in combination with type 2 diabetes vs patients with type 2 diabetes with no plaque vs glucose tolerant subjects [10]. This relationship with insulin resistance is further reported in many studies documenting that patients who showed impaired glucose tolerance or with diabetes also had decreased Omentin levels [11,12]. During weight loss due to diet or an exercise regimen, Omentin levels increased over time [13,14], further solidifying its link to obesity and body weight.

Omentin has also been linked to chronic inflammation, with reduced levels in rheumatoid arthritis, Crohn’s disease and asthma (reviewed in [1]). All these studies point towards a possible link between this visceral adipose tissue produced hormone and a diseased state.

*In vitro*, Omentin enhances insulin-stimulated glucose uptake in human adipocytes as well as activating Akt signaling pathways [3]. Pre-treatment of rat cells with Omentin significantly decreased TNF-α mediated phosphorylation of p38 and JNK, expression of VCAM-1, monocyte adhesion to smooth muscle cells, and NOX-derived superoxide production [4]. In adipose tissue explants, both insulin and glucose decrease Omentin mRNA expression and secretion [15]. To date, no receptor has been identified for intracellular transduction of these functions.

Severely obese patients have few options for weight loss and resolution of comorbidities that are as effective as bariatric surgery [16]. In fact, bariatric surgery was recently recognized as a valid treatment option for type 2 diabetes in obese patients by both the International Diabetes Federation and the American Diabetes Association. Further, long-term (6-year) remission rates post-gastric bypass reached up to 31% in a study by Brethauer et al. [17] and up to 72% in the Swedish Obese Subjects Study (SOS; with a follow-up of 2 years) [18], and result in improvements in cardiovascular function [19]. Biliopancreatic diversion (BPD) with or without duodenal switch (DS) is frequently used and has shown improvement in insulin and glucose metabolism in most patients [20]. As Omentin has demonstrated insulin-sensitizing effects and because patients undergoing BPD-DS become more insulin-sensitive mere days following surgery, even before any weight loss [21], Omentin levels were evaluated in a cohort of BPD-DS patients in the early stages post-surgery (1 day and 5 days, prior to weight loss) and over a longer period (6 months and 1 year, a period associated with weight loss). Specifically, we propose that Omentin levels will increase post-bariatric surgery and that this rise will precede gain of insulin sensitivity.

## Methods

### Subjects

Men and women >18 years of age were recruited into this study before undergoing biliopancreatic diversion (BPD) with duodenal switch (BPD-DS) at the Institut Universitaire de Cardiologie & Pneumologie de Québec (IUCPQ). Inclusion criteria were a BMI ≥40 kg/m^2^ or ≥35 kg/m^2^ with associated comorbidities. Patients who had previously undergone bariatric surgery or bore a pacemaker were excluded. Further, only patients for whom blood samples were taken at all 5 time points (pre-operative, day 1, day 5, 6 months and 1 year post-operatively) were included for further analysis. All laboratory measurements were finalized before statistical analyses were begun. All patients gave their informed, written consent; the protocol was approved by the ethics committee of the Research Center of IUCPQ, affiliated with Laval University.

### Anthropometric data

Height was measured with a stadiometer (SECA, 216 1814009, Brooklyn, NY). Weight, BMI, fat and lean masses were measured with an electrical bioimpedance balance (Tanita TBF-310, Tokyo, Japan) following a 12-hour fast. Medical history and treatments were collected from clinical file consultations.

### Plasma analyses

Blood samples were collected following a 12-hour fast into EDTA containing tubes and kept on ice until centrifugation (within 15 minutes). Plasma was then aliquoted into microtubes and stored at −80°C until analysis. Glycated hemoglobin (HbA1c) was measured in fresh samples by turbidimetric inhibition immunoassay. Standard clinical methodology (hospital biochemistry laboratory) was used to measure biochemical parameters while high-sensitive C-reactive protein (hsCRP) and apolipoprotein B (apoB) levels were measured by immunoturbidimetric method (Integra 800 System, Roche Diagnostics, IN, USA). All other biochemical assays were performed using a Modular system (Roche Diagnostics). LDL-cholesterol concentration was calculated using the Friedewald formula [22]. Homeostatic model assessment of insulin resistance (HOMA-IR) was calculated from fasting plasma insulin and glucose levels as (insulin × glucose)/22.5, where insulin concentration is converted from pmol/L to milliunits per liter and glucose as millimolar concentrations. Omentin was measured using Enzo Life Sciences (Farmingdale, NY, USA) ELISA which has a sensitivity of 0.4 ng/mL. Intra- and inter-assay CV% error are reported by the manufacturer as being <7.4% and <9.3%, respectively.

### Statistical analysis

Data are expressed as mean ± SEM as indicated. Student T or Chi^2^ tests were used to assess differences between individual variables or time points. For comparisons across multiple time points, repeated measures ANOVA with Holm-Sidak post-tests was used. Relationships between variables in each group were assessed by linear regression analysis using Spearman correlation. Prism (GraphPad Software Inc, La Jolla, CA, USA) and SigmaStat (Systat Software Inc., San Jose, CA, USA) software programs were used for graph and statistical analyses. Significance was set at p ≤ 0.05, where NS indicates “not statistically significant”.

## Results

### Pre-operative subject characteristics

As shown in Table 1, 18 men and 48 women undergoing BPD-DS were recruited in this study. Fifty percent of the patients (n = 33) had type 2 diabetes and 31 were receiving drug regimen before surgery. Subjects were an average of 46.7 ± 1.3 years old with a BMI of 49.6 ± 0.1 kg/m^2^. Average HOMA-IR was 8.81 ± 0.95, demonstrating generalized insulin resistance, as also indicted by elevated fructosamine (215 ± 4 μmol/L) and HbA1c (6.20 ± 0.01%) levels. Circulating hepatic and cardiac related risk markers were within normal clinical ranges (bilirubin, calcium, N-terminal pro-brain natriuretic peptide (NT-proBNP), gamma-glutamyl transpeptidase (GGT), and homocysteine). Red blood cell count, hemoglobin and hematocrit levels were within the normal range. Acylation Stimulating Protein (ASP; 32.2 ± 2.3 nmol/L) and IL-6 (8.19 ± 1.03 pg/mL) were also measured.

**Table 1:** **Pre-operative levels in all subjects**

**Variables**	**All subjects (n = 66) ± SEM**	**Range**	**Omentin-INC (n = 39) ± SEM**	**Omentin-DEC (n = 12) ± SEM**
Men/Women	18/48	-	12/27	2/10
Diabetic/Non-diabetic	33/33	-	19/20	5/7
T2D treatment	31/33 (94%)	-	15/24	5/7
Lipid lowering treatments	30/66 (45%)	-	15/24	3/9
Age (years)	46.7 ± 1.3	26.4 - 68.3	47.0 ± 1.7	46.6 ± 3.8
Weight (kg)	136 ± 3	94 - 205	134 ± 5	130 ± 6
BMI (kg/m^2^)	49.6 ± 0.1	35.9 - 75.5	48.8 ± 1.3	50.7 ± 1.7
Lean mass (kg)	65.8 ± 1.7	48.6 - 116.6	68.9 ± 3.1	68.8 ± 4.1
% fat mass	51.3 ± 0.7	33.8 - 66.8	51.0 ± 0.9	53.0 ± 0.9
Omentin (ng/mL)	11.0 ± 0.6	2.2 - 22.0	9.8 ± 0.8	11.7 ± 1.4
Glucose (mmol/L)	6.72 ± 0.29	4.20 - 16.00	6.52 ± 0.33	6.28 ± 0.96
Insulin (pmol/L)	195 ± 17	43 - 846	208 ± 25	200 ± 41
HOMA-IR	8.81 ± 0.95	1.51 - 39.91	9.16 ± 1.30	8.65 ± 2.68
Fructosamine (μmol/L)	215 ± 4	161 - 320	214 ± 5	211 ± 14
HbA1c (%)	6.20 ± 0.01	4.00 - 9.00	6.00 ± 0.16	0.061 ± 0.003
Cholesterol (mmol/L)	4.64 ± 0.11	2.72 - 7.53	4.60 ± 0.14	4.68 ± 0.17
Triglycerides (mmol/L)	1.63 ± 0.13	0.53 - 7.47	1.57 ± 0.15	1.40 ± 0.12
LDL (mmol/L)	2.64 ± 0.09	1.21 - 4.57	2.64 ± 0.11	2.60 ± 0.15
HDL (mmol/L)	1.29 ± 0.04	0.65 - 2.26	1.26 ± 0.05	1.39 ± 0.11
apoB (g/L)	0.77 ± 0.02	0.43 - 1.19	0.77 ± 0.03	0.80 ± 0.05
White blood cells (% total blood cell count)	8.10 ± 0.30	3.20 - 21.40	7.68 ± 0.26	8.20 ± 0.76
Neutrophils (% of white blood cells)	63.0 ± 1.00	46.0 - 60.0	62.3 ± 0.9	62.6 ± 2.3
Monocytes (% of white blood cells)	7.00 ± 0.20	3.00 - 12.00	6.75 ± 0.29	7.27 ± 0.80
Lymphocytes (% of white blood cells)	27.0 ± 1.00	24.0 - 35.0	27.8 ± 0.8	27.3 ± 2.0
Red blood cells (Total cell count ×10^12^/L)	4.60 ± 0.05	3.74 - 5.60	4.65 ± 0.06	4.44 ± 0.12
ASP (nmol/L)	32.2 ± 2.30	9.9 - 87.6	29.1 ± 2.8	31.2 ± 4.3
IL-6 (pg/mL)	8.19 ± 1.03	0.01 - 59.67	8.41 ± 1.68	6.84 ± 1.19
Hemoglobin (nmol/L)	136 ± 1	102 - 170	139 ± 2	132 ± 3
Total Bilirubin (μmol/L)	7.77 ± 0.42	3.00 - 23.00	7.39 ± 0.45	10.00 ± 1.43*
NT-proBNP (pg/mL)	50.9 ± 5.8	5.0 - 226.0	50.4 ± 7.9	78.6 ± 26.2
Homocysteine (μmol/L)	10.1 ± 0.5	5.3 - 25.0	10.1 ± 0.6	11.7 ± 1.4

*Abbreviations*: *BMI* body mass index, *WBC* white blood cells, * p = 0.025.

### Baseline Omentin levels correlate with white blood cell count and adipokines

**Figure 1:**
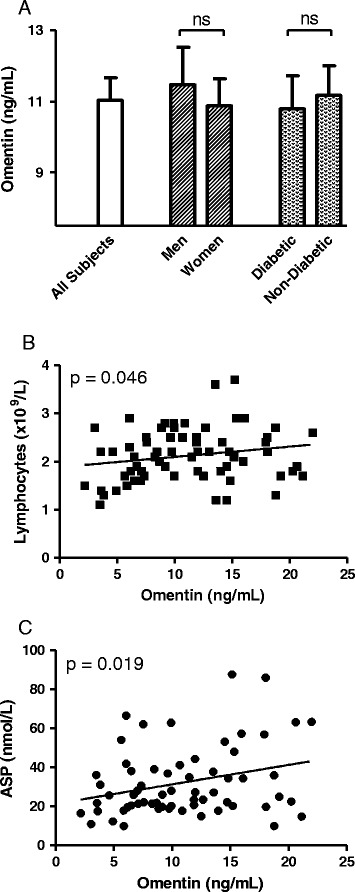
**Circulating Omentin levels and correlations in preoperative state.** Fasting Omentin levels in the preoperative state were measured in all subjects. **A**: Fasting Omentin levels separated based on sex and presence/absence of diabetes. Results are given as mean ± SEM. **B** and **C**: Correlation (Spearman) of Omentin levels to lymphocyte counts and circulating acylation stimulating protein (ASP).

Average preoperative Omentin levels were 11.0 ± 0.6 ng/mL, ranging from 2.2 to 22.0 ng/mL (Table 1). Pre-operative plasma Omentin levels were not significantly different in men vs women and were not affected by diabetic status (Figure 1A). Lymphocytes and ASP were positively correlated with Omentin (p = 0.046, Figure 1B and p = 0.019, Figure 1C, respectively).

### Omentin levels increase acutely post-surgery

**Figure 2:**
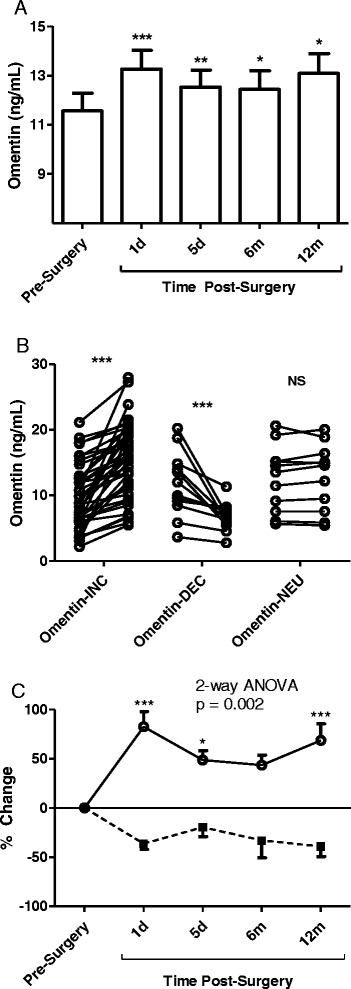
**Fasting pre-operative and post-operative Omentin levels. A**: Fasting levels of Omentin were measured pre-operative, and at 1 day, 5 days, 6 months and 12 months post-operatively. Results are presented as average ± SEM with analysis by ANOVA vs pre-operative levels. **B**: Pre-operative and 1 day levels of Omentin are given individually for subjects separated based on >10% increase (Omentin_INC_), >10% decrease (Omentin_DEC_), and no change in Omentin (neutral = Omentin_NEU_). **C**: Percent Omentin change across all time points is given for the Omentin_INC_ (solid line) and the Omentin_DEC_ (dotted line) group. Results are presented as average ± SEM with analysis by 2-way ANOVA. Significance is presented as *p < 0.05, **p < 0.01 and ***p < 0.001.

Figure 2 shows Omentin levels before and after BPD-DS surgery in the total group. A rapid increase of Omentin can be seen as early as 24 hours post-surgery and this increase is maintained up to 1 year after the surgery.

### Post-op Omentin levels differentiate into two groups

Of the 66 patients enrolled in the study, most (39 of 66; 59%) had Omentin levels that increased acutely at one-day following surgery (Omentin_INC_ Figure 2B, p < 0.0001 by paired *t*-test) while in 12 patients (18%) Omentin decreased >10% acutely following the surgery (Omentin_DEC_ Figure 2B, p < 0.0001 by paired *t*-test). A group of 15 patients (23%) showed no overall significant change in their Omentin levels at one-day post-surgery (p = NS by paired *t*-test). As these variations of Omentin were within the inter-assay variation coefficient of the ELISA (<10%), they were excluded from further analysis (Omentin-neutral group, Figure 2B). In those patients with acute changes at 1 day, these changes persisted throughout the follow-up, and were evident both at 5 days (prior to any change in weight) as well as long term (6 months and 12 months) with substantial weight loss. As seen in Figure 2C, although the groups are defined based on 1-DAY changes, they remain significantly different at each of the subsequent time periods as well as over the one year follow-up period (p = 0.002), with Omentin_INC_ patients maintaining higher Omentin levels throughout the 1-year period and Omentin_DEC_ patients also maintaining lower levels throughout.

### Omentin_INC_ and Omentin_DEC_ groups: acute 1-day changes in omentin levels correlate with long-term (1 year) health parameters

At baseline, subjects separated by acute (1-DAY) changes in Omentin were not significantly different in terms of pre-operative age, sex distribution, presence of diabetes or lipid lowering therapy, BMI, waist circumference, fat mass, or any of the fasting circulating variables measured as listed in Table 1, including glucose and lipid parameters (Table 1). Only total bilirubin was elevated in the Omentin_DEC_ group, 10.00 ± 1.43 μmol/L vs Omentin_INC_ 7.39 ± 0.45 μmol/L, (p = 0.025).

**Figure 3:**
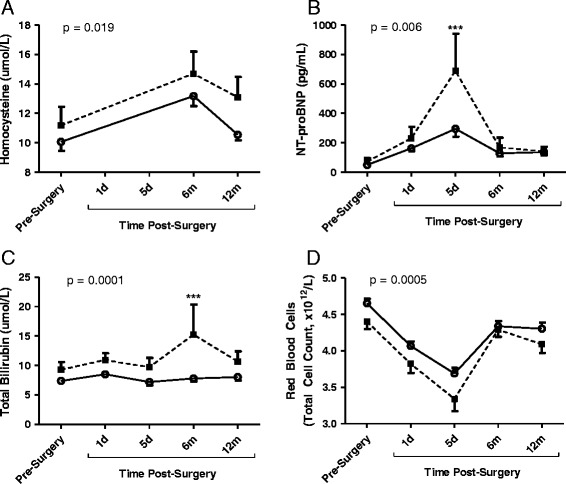
**Pre-operative and post-operative results for cardiovascular risk factors presented for groups based on Omentin**_**INC**_ **and Omentin**_**DEC**_ **.** Preoperative and postoperative results are presented for homocysteine **(A)**, NT-proBNP **(B)**, total bilirubin **(C)** and red blood cell counts **(D)** for the Omentin_INC_ (solid line) and Omentin_DEC_ (dotted line) groups over one year follow-up. Results are expressed as average ± SEM with analysis by 2-way ANOVA, significance for groups difference is indicated with post-hoc comparisons indicated: *** p < 0.001.

Subjects with Omentin levels that decreased 1-day after surgery had elevated levels of homocysteine (p = 0.019), NT-proBNP (p = 0.006) and total bilirubin (p = 0.0001) while red blood cell (RBC) count was lower (p = 0.0005) over the total study period (Figure 3). These differences between the two groups were not confounded by such parameters as weight (pre-surgery) or weight loss (up to 1 year after surgery) as all patients lost the same average amount of weight as shown by BMI (6-month BMI: Omentin_DEC_ group 36.72 ± 1.52 kg/m^2^ vs Omentin_INC_ 35.72 ± 1.02 kg/m^2^, p = ns and 12-month BMI: Omentin_DEC_ group 30.54 ± 1.37 kg/m^2^ vs Omentin_INC_ 30.72 ± 0.90 kg/m^2^, p = ns). Further, the resolution of diabetes within both groups was rapid and comparable.

### Weight and inflammation correlate overall with acute changes in Omentin levels

Baseline pre-surgery BMI inversely correlated (p = 0.008) with the acute 1-day changes in Omentin levels and this was reflected in both total weight and fat mass correlations (p = 0.048 and p = 0.037, respectively). As well, IL-6 levels before surgery also inversely correlated with the acute changes in Omentin (p = 0.02, Table 2).

**Table 2:** **Correlations between pre-operative variables and acute 1-DAY changes in Omentin**

**Pre-op parameters**	**% 1D OM**	**p=**
Weight (kg)	−0.263	0.048
Fat Mass (kg)	−0.277	0.037
BMI (kg/m^2^)	−0.347	0.008
IL-6 (pg/mL)	−0.316	0.020

Further, in the groups as a whole, levels of Omentin at 1-day following surgery correlated with many immune- and blood-related parameters. White blood cell count changes at 6 months and 1 year were inversely correlated with Day-1 Omentin levels (p = 0.016 and p < 0.001, respectively) as were neutrophil values (% total white cell count, p = 0.01 at 6 months and p = 0.022 at 1 year). Additionally, % monocytes and % lymphocytes correlated positively with Day-1 Omentin (p = 0.002, 1 year and p = 0.027, 6 months, respectively). One-year hemoglobin and total bilirubin levels at 6 months (p < 0.005 and p = 0.006, respectively) both correlated negatively as shown in Table 3.

**Table 3:** **One-day post-surgery levels of Omentin correlation with long-term health parameters**

	**∆183D**		**∆365D**	
**Variables**	**Correlation coefficient**	**p**	**Correlation coefficient**	**p**
White blood cell count (10^9^/L)	−0.322	0.016	−0.448	<0.001
% neutrophils (% of white blood cells)	−0.341	0.010	−0.301	0.022
% monocytes (% of white blood cells)	-	-	0.396	0.002
% lymphocytes (% of white blood cells)	0.296	0.027	-	-
Hemoglobin (nmol/L)	-	-	−0.361	0.005
Bilirubin, total (μmol/L)	−0.336	0.006	-	-

*Abbreviations*: *WBC* white blood cell.

## Discussion

The adipokine Omentin has been increasingly implicated in metabolic dysfunctions. Pre-surgery values of Omentin positively correlated with both BMI and ASP levels. This contrasts with observations in previous studies, where Omentin decreases with weight increases [3,5], however our correlations may be confounded by the subjects, which were all severely obese. In the present study, changes that occurred in Omentin levels following BPD-DS were evaluated both in the short-term as well as up to one year following surgery. As expected, subjects showed significant weight loss in the months following BPD-DS. In corroboration with other weight loss studies [14] we show that, in a large proportion of the patients, Omentin levels increase post-operatively. Interestingly, this change in Omentin occurred even before induction of weight loss, and these levels were maintained up to one-year post-bariatric intervention. Specifically, the Omentin increase occurred by one day post-surgery, even prior to resolution of the diabetic status, which occurred in many of these subjects by 5 days post-surgery [21]. However, not all patients demonstrated this Omentin increase following BPD-DS. In fact, 18% saw their levels decrease >10% by 24 hours after surgery but still demonstrated a resolution of their diabetic status in the short-term post-surgery. Further, these decreased levels were consistently maintained up to 1 year following the surgery.

Interesting differences could also be seen between the groups, Omentin_DEC_ and Omentin_INC_, at various time points. Omentin_DEC_ group was characterized by elevation of markers related to heart disease (both short- and long-term). Homocysteine, bilirubin and red blood cell counts were significantly different between the two groups throughout the one-year follow-up period, while NT-proBNP showed an overall difference but a peak difference at day 5 post-surgery. Increases in NT-proBNP have been linked to increased mortality rates in patients and several studies have proposed this as a marker of left ventricular diastolic dysfunction and heart failure [23–26], although in the present study the patients have not been followed up long enough, or with these outcomes as study criteria.

In line with these results, homocysteine increases are also linked to left ventricular diastolic function as well as coronary artery disease [27,28]. Levels of this non-protein α-amino acid can reflect vitamin intake, especially vitamin B6, which is needed as a cofactor for the transformation of homocysteine into cysteine [28,29]. Thus, in a cohort of patients receiving not only restrictive but also malabsorptive surgery, vitamin intake could well play a significant role in homocysteine levels. Patients receive high doses of vitamins to curtail any negative impact of bariatric surgery and although patient compliance can never be fully assured, vitamin A, D, B12 and folate levels remained within normal healthy parameters up to 1 year post-surgery (data not shown).

Elevated bilirubin levels have been shown to correlate with increased heart failure and death and corroborate the observations made with the previously mentioned biomarkers [30,25]. By contrast, other studies demonstrate that lower bilirubin levels correlate closely to coronary heart disease and metabolic syndrome components [31,32]. Further, bilirubin is the end-product of heme degradation which would be consistent with the observation of lowered red blood cell count in the Omentin_DEC_ group. Interestingly, bilirubin levels are elevated before the surgery in this group of patients (as well homocysteine), suggesting that these patients are already more at risk, even before any changes in diabetic status or Omentin levels.

Other studies have reported a link between Omentin and heart disease. Moreno-Navarette et al. suggested that Omentin, after controlling for adiposity, age, and inflammation in patients with impaired glucose tolerance, contributed independently to endothelial dysfunction [13]. A very recent study [33] shows that patients with lower levels of Omentin had increased risk of heart failure and even suggest its use as a novel prognostic marker for risk stratification in heart failure patients. This is further reinforced mechanistically by a study from Kataoka and colleagues [34], where systemic injection of Omentin in mice that suffered sustained ischemic injury led to a reduction of myocardial infarct size and apoptosis. Further, studies have also shown lower Omentin levels in patients with type 2 diabetes compared to healthy controls and even lower Omentin levels when patients with diabetes presented with carotid plaque [10].

While the functions of Omentin are still being elucidated and its receptor is still unknown, a role as “insulin sensitizer” in adipocytes has already been demonstrated. However, recent evidence supports the potential for an as yet unidentified role in heart function based on the links to heart disease. Interestingly, pre-operative BMI (as well as weight and fat mass) correlated negatively with the acute (1 day) change in Omentin, as did IL-6 levels, perhaps indicating that a subgroup of these severely obese patients are already at-risk for the development of cardiovascular problems and this could be associated with Omentin (and other factors) in an as-yet-undiscovered function. Initial studies on Omentin (then known as Intelectin) focused on its microbial surface galactofuranose binding properties. Thus, it was suggested to participate in host early-defense against bacterial infections [35]. Literature in recent years has shown the importance of gut microbiota in the development of obesity as well as insulin resistance and other metabolism-related comorbidities (as reviewed in [36]). We would speculate that Omentin could be part of this link, being produced by visceral adipose tissue and acting as an “insulin sensitizer” yet also being able to interact with specific bacteria in the gut. BPD-DS also plays a role in post-surgery microbiota redistribution (reviewed in [37]) and this could possibly be evidenced through changes in Omentin levels. Further, Omentin has demonstrated anti-inflammatory properties, cementing its links with CVD and Metabolic Syndrome (reviewed in Jaikanth et al. [1]. Thus, lower levels of this adipokine could well be implicated in worsening of situations where immune activation is present, as is the case in obesity and diabetes. These proposed roles of Omentin, and correlations with ASP and lymphocytes, mesh with the concept of the contribution of various biomarkers to an enhanced pro-inflammatory state and autoimmune activation as pivotal in understanding chronic diseases, as reviewed in detail by Onat et al. [38].

The limitations of this present study are that all patients were severely obese, and as such interpretations cannot be generalized to other groups. Further, given the exploratory nature of Omentin physiology, with little known regarding regulation, signaling pathway, receptor target and broader functions in various tissues, the current correlations cannot be interpreted as causative. Further studies will be necessary to determine the possible links between Omentin and risk of heart failure and whether this novel adipokine could be used as an early biomarker to identify patients with increased risk, and whether special care or treatment should be administered.

## Conclusions

Altogether, the present study suggests that a subgroup of patients (approximately 25%) following bariatric surgery may be at greater risk for heart-related complications, at least based on the presentation of known risk markers, both preoperatively and within the one year follow-up period following surgery. Further, Omentin levels 1 day following surgery may be a marker of this “higher risk” group, where lowered Omentin correlates with higher levels of risk factors overall, well before any weight loss or changes in adiposity and before insulin sensitivity is restored.

## Abbreviations

ApoB: Apolipoprotein B
ASP: Acylation stimulating protein
BMI: Body mass index
BPD: Biliopancreatic diversion
DS: Duodenal switch
GGT: Gamma-glutamyl-transpeptidase
HOMA-IR: Homeostatic model assessment index-insulin resistance
HbA1c: Hemoglobin A1c
hsCRP: High-sensitivity C-reactive protein
IL-6: Interleukin-6
LDL-C: Low density lipoprotein-cholesterol
NT-proBNP: N-terminal pro-brain natriuretic peptide
RBC: Red blood cells
